# Do Epigenetic Events Take Place in the Vastus Lateralis of Patients with Mild Chronic Obstructive Pulmonary Disease?

**DOI:** 10.1371/journal.pone.0102296

**Published:** 2014-07-11

**Authors:** Ester Puig-Vilanova, Pilar Ausin, Juana Martinez-Llorens, Joaquim Gea, Esther Barreiro

**Affiliations:** 1 Pulmonology Department-Muscle and Respiratory System Research Unit (URMAR), IMIM-Hospital del Mar, Parc de Salut Mar, Health and Experimental Sciences Department (CEXS), Universitat Pompeu Fabra (UPF), Barcelona Biomedical Research Park (PRBB), Barcelona, Spain; 2 Centro de Investigación en Red de Enfermedades Respiratorias (CIBERES), Instituto de Salud Carlos III (ISCIII), Madrid, Spain; Universidad Pablo de Olavide, Centro Andaluz de Biología del Desarrollo-CSIC, Spain

## Abstract

Muscle dysfunction is a major comorbidity in Chronic Obstructive Pulmonary Disease (COPD). Several biological mechanisms including epigenetic events regulate muscle mass and function in models of muscle atrophy. Investigations conducted so far have focused on the elucidation of biological mechanisms involved in muscle dysfunction in advanced COPD. We assessed whether the epigenetic profile may be altered in the vastus lateralis of patients with mild COPD, normal body composition, and mildly impaired muscle function and exercise capacity. In vastus lateralis (VL) of mild COPD patients with well-preserved body composition and in healthy age-matched controls, expression of DNA methylation, muscle-enriched microRNAs, histone acetyltransferases (HTAs) and deacetylases (HDACs), protein acetylation, small ubiquitin-related modifier (SUMO) ligases, and muscle structure were explored. All subjects were clinically evaluated. Compared to healthy controls, in the VL of mild COPD patients, muscle function and exercise capacity were moderately reduced, DNA methylation levels did not differ, miR-1 expression levels were increased and positively correlated with both forced expiratory volume in one second (FEV_1_) and quadriceps force, HDAC4 protein levels were increased, and muscle fiber types and sizes were not different. Moderate skeletal muscle dysfunction is a relevant feature in patients with mild COPD and preserved body composition. Several epigenetic events are differentially expressed in the limb muscles of these patients, probably as an attempt to counterbalance the underlying mechanisms that alter muscle function and mass. The study of patients at early stages of their disease is of interest as they are a target for timely therapeutic interventions that may slow down the course of the disease and prevent the deleterious effects of major comorbidities.

## Introduction

Chronic Obstructive Pulmonary Disease (COPD) is a highly prevalent condition that represents a major cause of death in developed countries. COPD also imposes a substantial economic burden in the health care systems as patients may require long hospital stays or costly therapeutic interventions for acute exacerbations. Comorbidities such as skeletal muscle dysfunction with and without muscle loss are characteristic features of patients with COPD even at early stages of their disease [1]–[4]. Muscle dysfunction is defined as the impairment of one of the two main properties of muscles, strength and endurance. In COPD, quadriceps muscle weakness, defined as reduced muscle force, and muscle mass loss were shown to predict the patients' survival and mortality [1], [2].

Several factors and mechanisms have been shown to participate in the etiology of COPD muscle dysfunction [2], [5]–[12]. Oxidative stress, systemic inflammation, structural abnormalities, mitochondrial derangements, autophagy, muscle wasting, and deconditioning are the most relevant biological contributors to COPD muscle dysfunction [2], [5]–[17]. Recently, epigenetic events have also emerged as potential regulators of muscle mass and function in the lower limbs of patients with severe COPD [18]–[21]. Epigenetic control of cells is the process whereby gene expression is regulated by heritable mechanisms that do not affect DNA sequence. It plays a major role in muscle adaptation to environmental factors such as immobilization, exercise, and muscle mass plasticity in several experimental models [22]–[26]. Furthermore, the expression of noncoding single-stranded RNA molecules, microRNAs (miRNAs) specific to skeletal muscles, was shown to be modified in limb muscles and blood of patients with severe COPD [19], [20]. Interestingly, in those patients, significant positive correlations were also found between expression levels of muscle-specific miRNAs and body composition [19], [20]. DNA methylation is a biochemical process characterized by the addition of a methyl group to the 5 position of the cytosine that stands before a guanine molecule in the same chain. DNA methylation is the most stable modification of chromatin that may vary during development and aging in cells. It determines cell fate and experiments have shown that demethylation of DNA in mesenchymal cells led to the development of several cell types including myocytes [27]. Nonetheless, whether DNA methylation plays a role in muscle adaptation to environmental factors in adult muscles or in COPD muscle dysfunction remains unknown.

Moreover, histone acetylation, defined as the balance between histone acetyltransferases (HTAs) and histone deacetylases (HDACs) also seemed to regulate muscle plasticity in response to environmental factors including muscle mass maintenance in a variety of models [28]–[31]. In keeping with, in the vastus lateralis of patients with advanced COPD and cachexia, total protein acetylation levels were increased, while protein content of HDAC3 and sirtuin-1 were reduced in those muscles (unpublished observations). Additionally, in the diaphragm of patients with moderate-to-severe COPD, a rise in HDAC4 levels was observed together with a decrease in several muscle-enriched miRNAs (unpublished observations). Collectively, the reported findings suggest that epigenetic events seem to play a prominent role in muscle adaptations to environmental factors such as deconditioning and muscle mass loss in several models including severe COPD. Nevertheless, results reported so far have been based on the analysis of muscles obtained from patients with severe conditions. Little information is currently available on the molecular and cellular mechanisms that are involved in the peripheral muscle dysfunction of patients with less advanced COPD, let alone the implications of epigenetic events in this comorbidity.

In muscular dystrophies and sarcopenia, increased levels of small ubiquitin-related modifier (SUMO) ligases were shown to underlie muscle wasting through premature senescence of satellite cells in experimental models [32], [33]. Whether this mechanism could also precipitate muscle dysfunction and mass loss remains to be identified in COPD.

On these grounds, we hypothesized that the epigenetic profile may be altered in the vastus lateralis of patients with mild COPD and normal body composition who already exhibited impaired muscle function and exercise capacity. Accordingly, the study objectives were as follows: 1) to assess DNA methylation and miRNA expression levels of muscle-specific and non-muscle specific but abundantly expressed in muscles, protein acetylation events, myogenic transcription factors, and SUMO-2/3 in the vastus lateralis of patients with mild COPD and preserved body composition, 2) to identify muscle fiber type composition and sizes, and 3) to analyze potential correlations between the different study variables among the patients. Specimens from the vastus lateralis were also obtained in sedentary controls for the purpose of the investigation, and both patients and control subjects were clinically and functionally evaluated.

## Methods

(See File S1 for detailed information on all the methodologies).

### Study subjects

Thirteen patients with stable COPD and 13 age-matched sedentary controls were recruited. Specimens from the vastus lateralis were obtained from all subjects on an out-patient basis. COPD patients were recruited from the COPD [34]–[37] Clinic at Hospital del Mar (Barcelona) and the control subjects were recruited from the general population (patients' relatives or friends) at Hospital del Mar. Smoking history was similar between patients and healthy controls. All patients were on bronchodilators. They were clinically stable at the time of the study, without episodes of exacerbation or oral steroid treatment in the previous four months. None of them presented significant comorbidities. All groups of individuals were Caucasian. Moreover, in the present investigation, the sedentary control subjects were also involved in another study aimed at investigating the epigenetic profile in limb muscles of patients with severe COPD and muscle wasting (unpublished observations, submitted).

Exclusion criteria for COPD patients and control subjects included other chronic respiratory (asthma) or cardiovascular disorders, acute exacerbations in the last 3 months, limiting osteoarticular condition, chronic metabolic diseases including diabetes, suspected para-neoplastic or myopathic syndromes, and/or treatment with drugs known to alter muscle structure and/or function including systemic corticosteroids. COPD patients and healthy controls were qualified as sedentary after being specifically inquired about whether they were conducting any regular outdoor physical activity, going regularly to the gymnasium, or participating in any specific training program.

The current investigation was designed in accordance with both the ethical standards on human experimentation in our institutions and the World Medical Association guidelines (Helsinki Declaration of 2008) for research on human beings. Approval was obtained from the institutional Ethics Committees on Human Investigation (*Hospital del Mar*, Barcelona). Informed written consent was obtained from all individuals.

### Anthropometrical and Functional Assessment

Anthropometrical evaluation included BMI and determination of the FFMI by bioelectrical impedance [10]. Nutritional parameters were also evaluated through conventional blood tests. Lung function was evaluated through determination of spirometric values, static lung volumes, diffusion capacity, and blood gases using standard procedures [38]–[40]. Quadriceps muscle strength was evaluated in both patients and controls by isometric maximum voluntary contraction (QMVC) of the dominant lower limb as formerly described [4], [41].

### Muscle biopsies and blood samples

#### Vastus lateralis biopsies

Muscle samples were obtained from the quadriceps muscle (vastus lateralis) of both groups of patients and control subjects using the open muscle biopsy technique, as described previously [7], [10]. Samples were 60–80 mg size in average.

Muscle sample specimens were always cleaned out of any blood contamination with saline. They were immediately frozen in liquid nitrogen and stored in the −80°C freezer (under permanent alarm control) for further analysis or immersed in an alcohol-formol bath for 2 h to be thereafter embedded in paraffin. Frozen tissues were used for immunoblotting techniques, while paraffin-embedded tissues were used for the assessment of myosin heavy chain isoforms (immunohistochemical analysis). All subjects were prevented from doing any potentially exhausting physical exercise 10 to 14 days before coming to the hospital to undergo the surgical procedures.

Blood samples were drawn at 8:00 am after an overnight fasting period in both patients and healthy controls.

### Molecular biology analyses

#### DNA isolation

Total DNA was isolated from vastus lateralis muscle of all study subjects using QIAmp DNA Mini Kit (QiAgen, Redwood City, CA, USA) [42], following the manufacturer's protocol of DNA purification from tissues, and without the use of RNase A. Total DNA obtained from muscles was quantified using a spectrophotometer (NanoDrop, Thermo Scientific, Wilmington, DE, USA).

#### Quantification of Methylated DNA using enzyme-linked immunosorbent assay (ELISA)-based immunoassay

Global 5-methylcytosine (5-mC) in DNA was quantified in the vastus lateralis of both patients and healthy controls using MethylFlash Methylated DNA Quantification Colorimetric Kit (Epigentek, Farmingdale, NY, USA) following the precise manufacturer's instructions and previous studies [43]. The minimum detectable concentration of methylated DNA in the samples was set to be 0.2 ng of methylated DNA. Data are expressed as the percentage of total methylated DNA to total DNA in the muscle samples.

#### RNA isolation

Total RNA was first isolated from snap-frozen skeletal muscles using Trizol reagent following the manufacturer's protocol (Life technologies, Carlsbad, CA, USA). Total RNA concentrations were determined photometrically using the NanoDrop 1000 (Thermo Scientific, Waltham, MA, USA).

#### MicroRNA and mRNA reverse transcription (RT)

MicroRNA RT was performed using TaqMan microRNA assays (Life Technologies) following the manufacturer's instructions. First-strand cDNA was generated from mRNA using oligo(dT)_12–18_ primers and the Super-Script III reverse transcriptase following the manufacturer's instructions (Life technologies).

#### Real time-PCR amplification (qRT-PCR)

TaqMan based qPCR reactions were performed using the ABI PRISM 7900HT Sequence Detector System (Applied BioSystems, Foster City, CA, USA) together with a commercially available predesigned microRNA assay, primers, and probes as shown in Tables 1 and 2. Taqman microRNA assay for small nuclear RNA U6 (snU6) was used to normalize the miRNAs amplifications, whereas the housekeeping gene glyceraldehyde-3-phosphate dehydrogenase (GAPDH) served as the endogenous control for mRNA gene expression. MicroRNA and mRNA data were collected and subsequently analyzed using the SDS Relative Quantification Software version 2.1 (Applied BioSystems), in which the comparative C_T_ method (2^−ΔΔCT^) for relative quantification was employed [44]. Results in the figures are expressed as the expression of fold change relative to mean value of the control group, which was equal to 1.

**Table 1:** MicroRNA assays used for the quantitative analyses of the target genes using real-time PCR.

Assay Name	Assay ID	miRBase accession number
**Muscle-specific, myomiRs**		
hsa-miR-1	002222	MIMAT0000416
hsa-miR-133a	002246	MIMAT0000427
hsa-miR-206	000510	MIMAT0000462
**Other miRNAs (highly expressed in muscles)**		
hsa-miR-486	001278	MIMAT0002177
hsa-miR-27a	000408	MIMAT0000084
hsa-miR-29b	000413	MIMAT0000100
hsa-miR-181a	000480	MIMAT0000256
		**NCBI Accession number**
U6 snRNA, housekeeping gene	001973	NR_004394

*Abbreviations*: ID, identification; hsa, homo sapiens; miR, microRNA; MIMAT, mature microRNA; snRNA, small nuclear RNA; and NR, non-coding RNA RefSeq database category.

**Table 2:** Probes used for the quantitative analyses of the target genes using real-time PCR.

Gene Symbol	Assay ID	Taqman probe context sequence (5′-3′)	Genbank accession number
EP300	Hs00914223_m1	CACCATGGAGAAGCATAAAGAGGTC	NM_001429.3
SUMO2	Hs02743873_g1	CATTGTAAAACCAAGGACAATTTTA	NM_006937.3
SUMO3	Hs00739248_m1	GCGAGAGGCAGGGCTTGTCAATGAG	NM_006936.2
GAPDH	Hs99999905_m1	GGGCGCCTGGTCACCAGGGCTGCTT	NM_002046.4

*Abbreviations*: ID, identification; EP300, E1A binding protein p300; Hs, homo sapiens; m1, multi-exonic gene assay does not detect genomic DNA; NM, mRNA RefSeq database category; SUMO, small ubiquitin-like modifier; g1, multi-exonic gene assay may detect genomic DNA if present in the sample; and GAPDH, glyceraldehyde-3-phosphate dehydrogenase.

#### Immunoblotting of 1D electrophoresis

Protein levels of the different molecular markers analyzed in the study were explored by means of immunoblotting procedures as previously described [5], [7], [10], [45], [46]. Protein levels of HDACs, HATs, and myogenic transcription factors were identified using specific primary antibodies: HDAC3 (anti-HDAC3 antibody, Santa Cruz Biotechnology, Santa Cruz, CA, USA), HDAC6 (anti-HDAC6 antibody, Epigentek, Farmingdale, NY, USA), HDAC4 (anti-HDAC4 antibody, Santa Cruz), NAD-dependent protein deacetylase sirtuin-1 (SIRT1) (anti-SIRT1 antibody, ProteinTech Group Inc., Chicago, IL, USA), myocyte enhancer factor (MEF)2C (anti-MEF2C antibody, Santa Cruz), MEF2D (anti-MEF2D antibody, Santa Cruz), YinYang (YY)1 (anti-YY1 antibody, Santa Cruz), and vinculin (anti-vinculin antibody, Santa Cruz). Antigens from all samples were detected with horseradish peroxidase (HRP)-conjugated secondary antibodies and a chemiluminescence kit. For each of the antigens, samples from the different groups were always detected in the same picture under identical exposure times.

PVDF membranes were scanned with the Molecular Imager Chemidoc XRS System (Bio–Rad Laboratories, Hercules, CA, USA) using the software Quantity One version 4.6.5 (Bio–Rad Laboratories). Optical densities of specific proteins were quantified using the software Image Lab version 2.0.1 (Bio-Rad Laboratories). In order to validate equal protein loading among various lanes, SDS-PAGE gels were stained with Coomassie Blue, and the cytoskeletal protein vinculin (117 kDa, Figure S1 in File S1) was used as the protein loading controls in all the immunoblots. Final optical densities obtained in each specific group of subjects corresponded to the mean values of the different samples (lanes) of each of the antigens studied, which were normalized to the optical densities of the loading control for each antigen and experimental group.

#### Muscle fiber counts and morphometry

On 3-micrometer muscle paraffin-embedded sections from vastus lateralis muscles of all study groups, MyHC-I and –II isoforms were identified using anti-MyHC-I (clone MHC, Biogenesis Inc., Poole, England, UK) and anti-MyHC-II antibodies (clone MY-32, Sigma, Saint Louis, MO), respectively, as published elsewhere [7], [10], [46]. The cross-sectional area, mean least diameter, and proportions of type I and type II fibers were assessed using a light microscope (Olympus, Series BX50F3, Olympus Optical Co., Hamburg, Germany) coupled with an image-digitizing camera (Pixera Studio, version 1.0.4, Pixera Corporation, Los Gatos, CA, USA) and a morphometry program (NIH Image, version 1.60, Scion Corporation, Frederick, MD, USA). At least 100 fibers were measured and counted in each muscle specimen from both study groups.

### Statistical Analysis

Data are expressed as mean (standard deviation). Comparisons of physiological, clinical, molecular and structural variables between the two study groups were analyzed using the Student's *T- test*. Correlations between clinical, physiological and biological variables were explored using the Pearson's correlation coefficient among both groups of patients. A level of significance of *P*≤0.05 was established.

The sample size chosen was based on previous studies [5]–[7], [10], [19]–[21], [45]–[49], where very similar approaches were employed and on assumptions of 80% power to detect an improvement of more than 20% in measured outcomes at a level of significance of *P*≤0.05.

## Results

### Clinical characteristics

Table 3 illustrates all clinical and functional variables of controls and COPD patients recruited in the study. No significant differences were observed in age, smoking history, or body composition between patients and control subjects. COPD patients exhibited mild airflow limitation and diffusion capacity impairment. Exercise capacity as measured by six-minute walking distance and cycloergometry, and muscle strength were mildly decreased in the COPD patients compared to healthy controls. Levels of fibrinogen and globular sedimentation velocity were moderately increased in the patients compared to controls.

**Table 3:** Main clinical characteristics and functional variables of all the study subjects.

	Controls	Mild COPD
	N = 13	N = 13
**Anthropometry**		
Age (years)	67 (5)	70 (6)
BMI (kg/m^2^)	25 (3)	25 (3)
FFMI (kg/m^2^)	18 (2)	18 (1)
**Smoking History**		
Active, N, %	6, 46	7, 54
Ex-smoker, N, %	4, 31	6, 46
Never smoker, N, %	3, 23	0, 0
Pack/year	53 (20)	50 (22)
**Lung function**		
FEV_1_ (% pred)	90 (11)	72 (4)***
FVC (% pred)	92 (11)	80 (7)**
FEV_1_/FVC (%)	72 (4)	62 (5)***
RV (% pred)	105 (11)	121 (28)
TLC (% pred)	104 (11)	101 (9)
RV/TLC (%)	44 (5)	48 (10)
DLco (% pred)	91 (15)	76 (15)*
K_CO_ (% pred)	90 (15)	73 (11)**
PaO_2_ (kPa)	11.6 (1.2)	11.1 (1.5)
PaCO_2_ (kPa)	5.5 (0.4)	5.2 (0.3)
**Exercise capacity & muscle force**		
VO_2_ peak (% pred)	92 (14)	74 (10)*
W*R* peak (% pred)	96 (18)	76 (8)*
Six-min walking test (m)	517 (57)	426 (17)***
QMVC (kg)	38 (2)	36 (1)***
**Blood parameters**		
Albumin (g/dL)	4.2 (0.4)	4.1 (0.5)
Total proteins (g/dL)	7.2 (0.5)	6.9 (0.8)
CRP (mg/dL)	0.3 (0.2)	0.4 (0.2)
Fibrinogen (mg/dL)	309 (36)	350 (50)*
GSV (mm/h)	5 (4)	13 (11)*

Values are expressed as mean (standard deviation).

*Abbreviations*: COPD, chronic obstructive pulmonary disease; N, number of patients; m, meters; BMI, body mass index; FFMI, fat-free mass index; kg, kilograms; FEV_1_, forced expiratory volume in one second; pred, predicted; FVC, forced vital capacity; RV, residual volume; TLC, total lung capacity; DLco, carbon monoxide transfer; K_CO_, *Krough* transfer factor; PaO_2_, arterial oxygen partial pressure; PaCO_2_, arterial carbon dioxide partial pressure; VO_2_ peak, peak exercise oxygen uptake; W*R* peak, peak work rate; QMVC, quadriceps maximal velocity contraction; g, grams; dL, deciliter; mg, miligrams.; CRP, C-reactive protein; GSV, globular sedimentation velocity; mm, millimeters; h, hour.

*Statistical significance*:

*, p≤0.05,

**, p≤0.01,

***, p≤0.001 between mild COPD patients and control subjects.

### Muscle biological markers

#### DNA methylation

**Figure 1:**
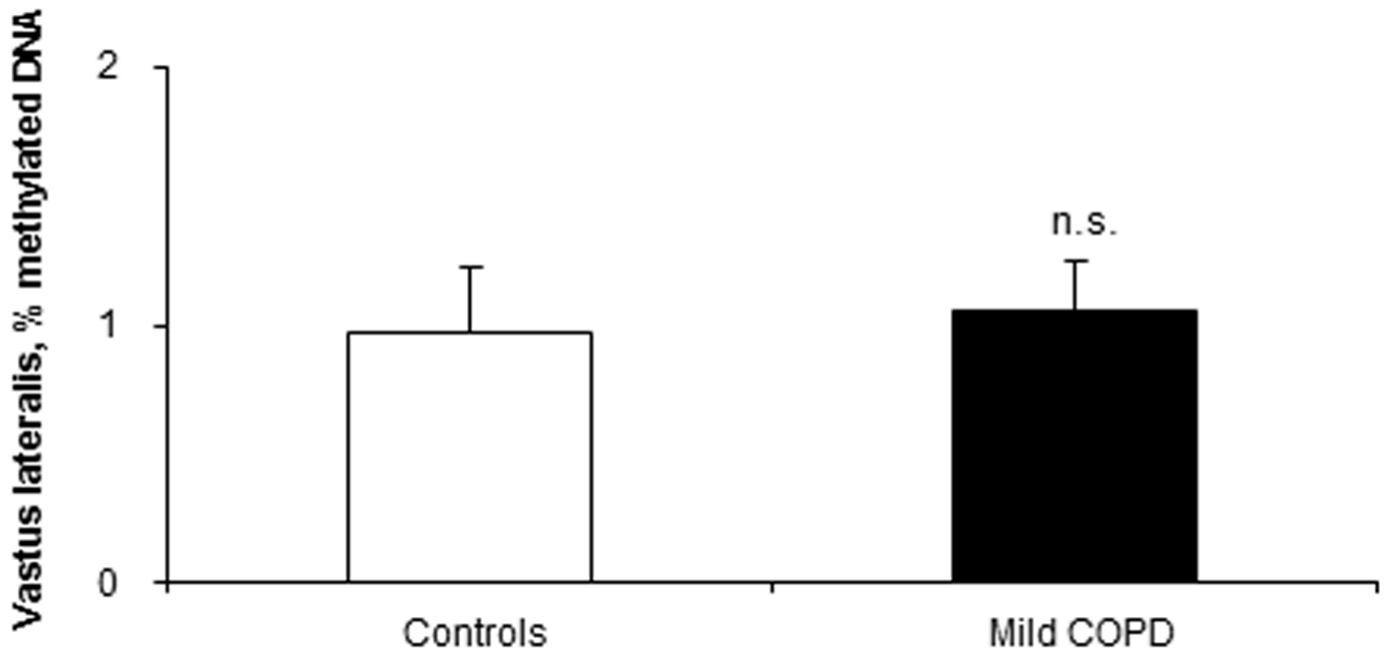
Global percentage of methylated DNA in the vastus lateralis of mild COPD patients and healthy controls. Mean values and standard deviation of global percentage of methylated DNA in the vastus lateralis did not differ (n.s., non-significant) between patients and controls.

The percentage of muscle methylated DNA to total DNA did not differ between patients and control subjects (Figure 1).

#### MicroRNAs expression

**Figure 2:**
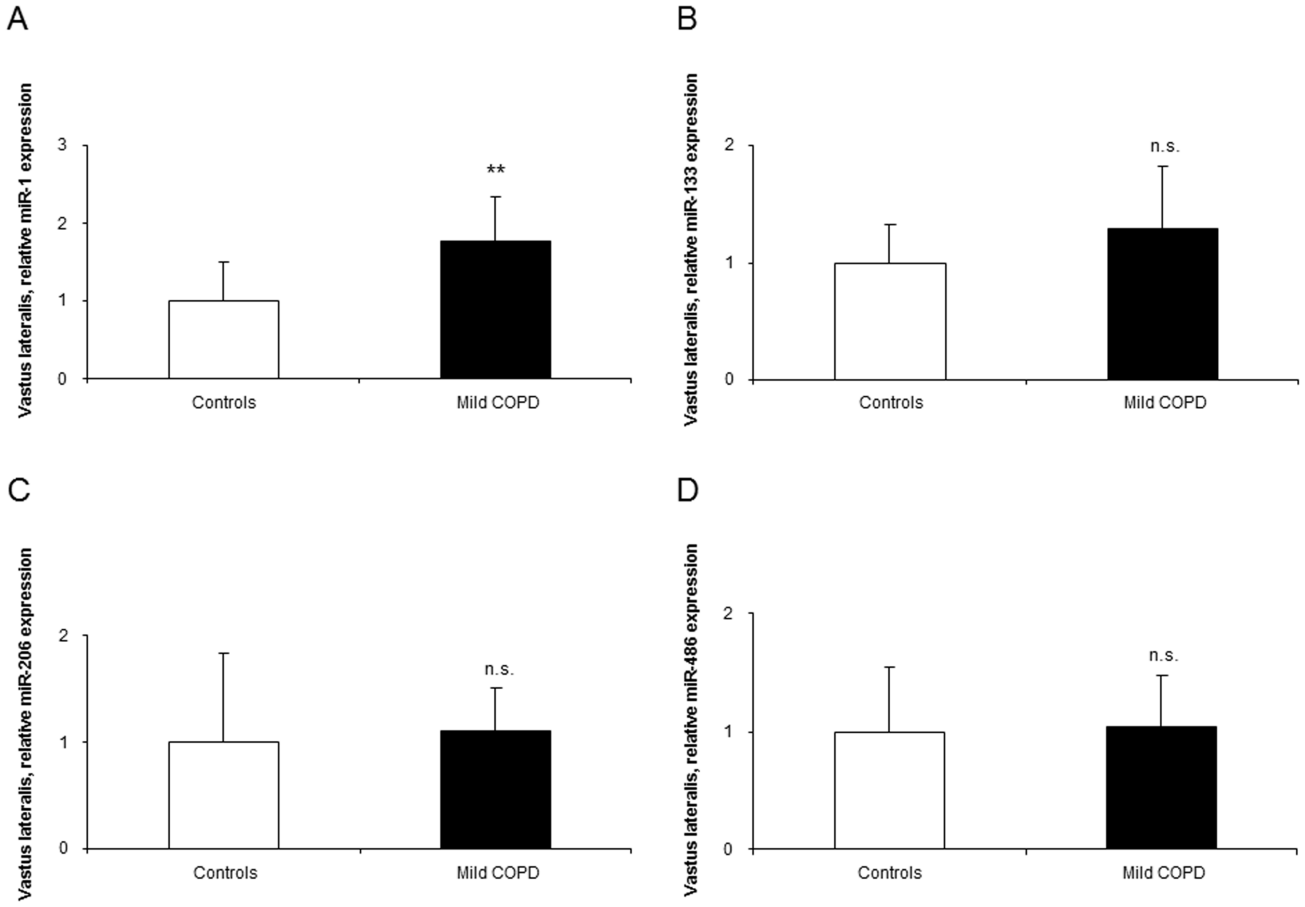
Levels of expression of muscle-enriched microRNAs in the vastus lateralis of mild COPD patients and healthy controls. Mean values and standard deviation (relative expression) of miR-1 (A) expression was upregulated (**: p<0.01) in the vastus lateralis of mild COPD patients compared to healthy controls, whereas expression levels of miR-133 (B), miR-206 (C), and miR-486 (D) did not differ (n.s., non-significant) in the lower limb muscles between the study groups.

**Figure 3:**
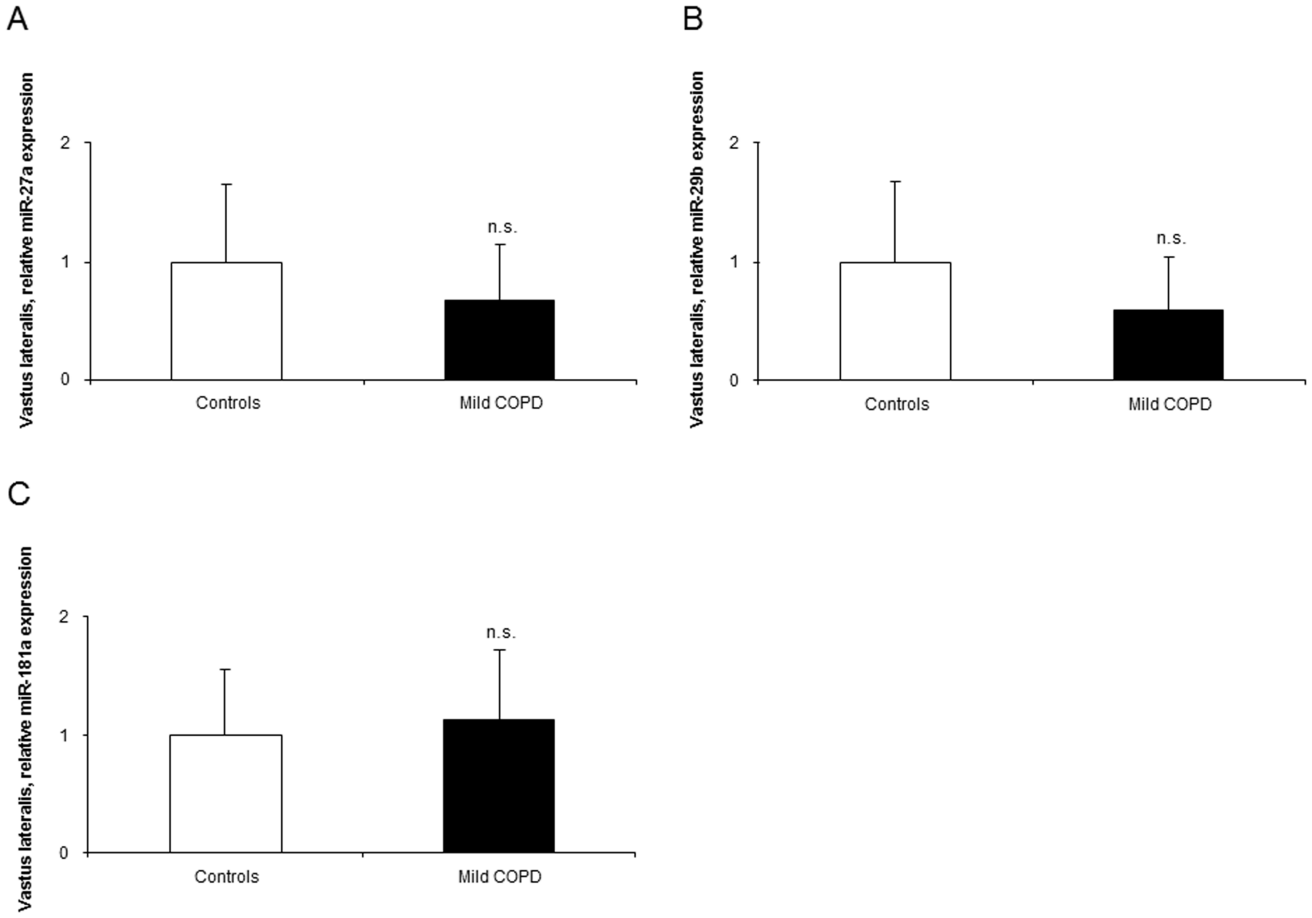
Levels of expression of muscle-enriched microRNAs in the vastus lateralis of mild COPD patients and healthy controls. Mean values and standard deviation (relative expression) of miR-27a (A), miR-29b (B), and miR-181a (C) did not differ (n.s., non-significant) in the lower limb muscles between the study groups.

**Figure 4:**
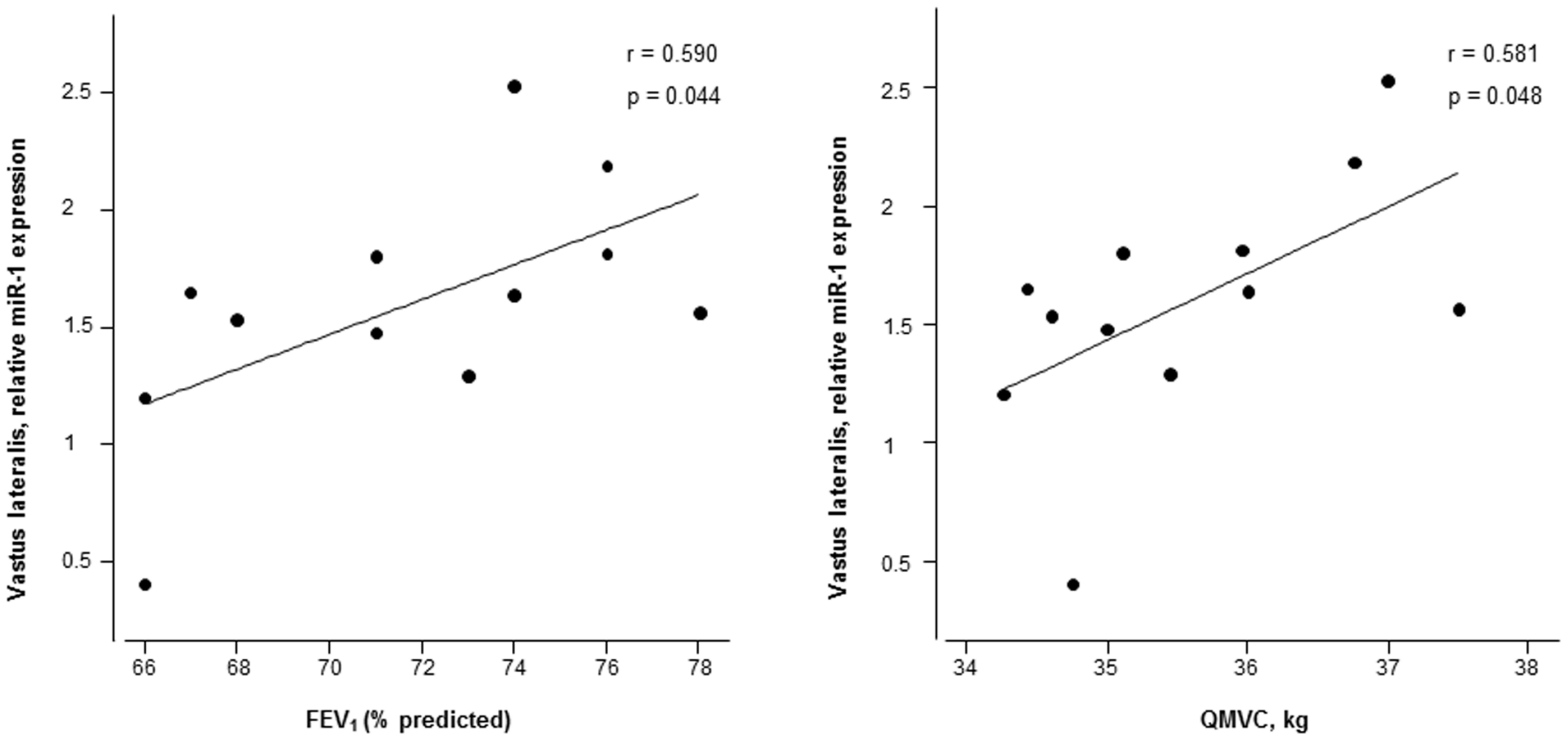
Significant correlations between expression levels of miR-1 in the muscles of the COPD patients and both FEV_1_ and QMVC. Among the COPD patients, significant positive correlations were observed between the physiological parameters FEV_1_ and QMVC and expression levels of miR-1 in their vastus lateralis. Note that only 12 patients were plotted in the graphs as miR-1 expression levels could not be obtained for technical reasons in one of the patients.

Compared to healthy controls, expression levels of miR-1 were significantly greater in the vastus lateralis of the patients than in the control subjects (Figure 2A). Nevertheless, the expression of miR-133, -206, -486, -27a, -29b, and -181a did not significantly differ between patients and controls (Figures 2B–D, and 3A–3C). Among the patients, significant positive correlations were found between muscle miR-1 expression levels and both the degree of airway obstruction (FEV_1_) and quadriceps muscle force (Figure 4). Additionally, positive correlations were also observed between miR-1 expression levels and those of miR-133 (r = 0.809, p = 0.001) and miR-486 (r = 0.712, p = 0.009) in the vastus lateralis of the patients.

#### Histone modifications

**Figure 5:**
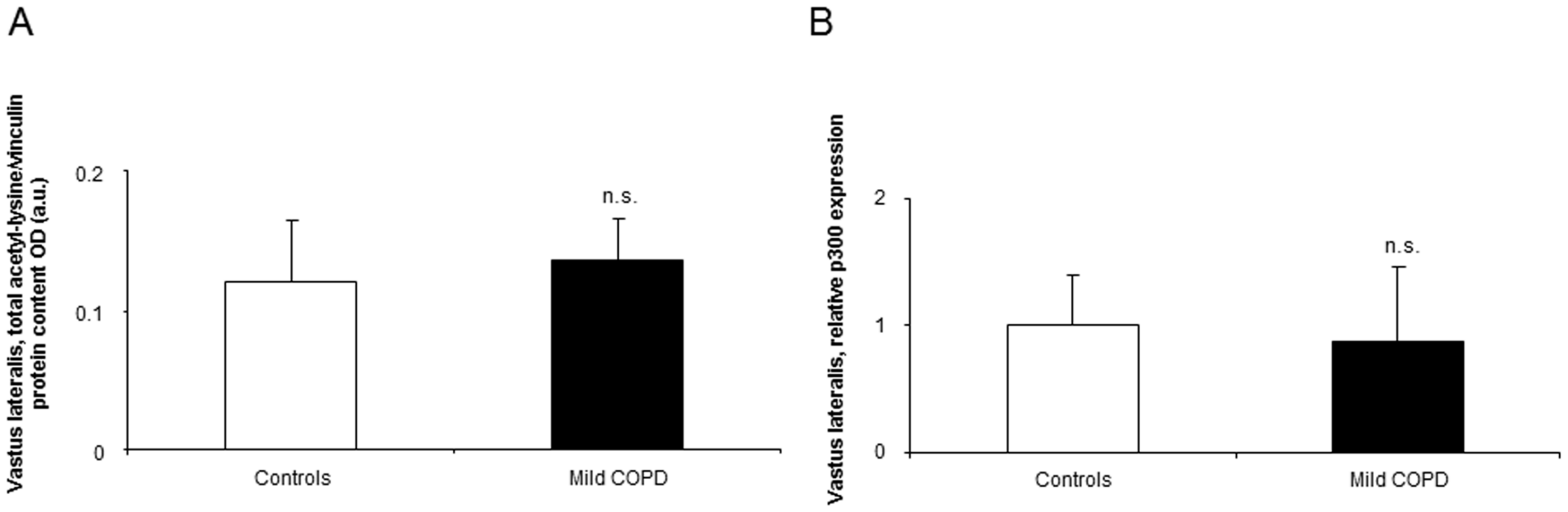
Protein levels of total acetyl-lysine protein content and mRNA levels of the HTA p300 in muscles of both COPD patients and controls. Mean values and standard deviation of total protein acetyl-lysine/vinculin protein loading control as measured in optical densities (OD) using arbitrary units (a.u.) (A) and HTA p300 (B), levels in the vastus lateralis did not differ (n.s., non-significant) between the two study groups.

**Figure 6:**
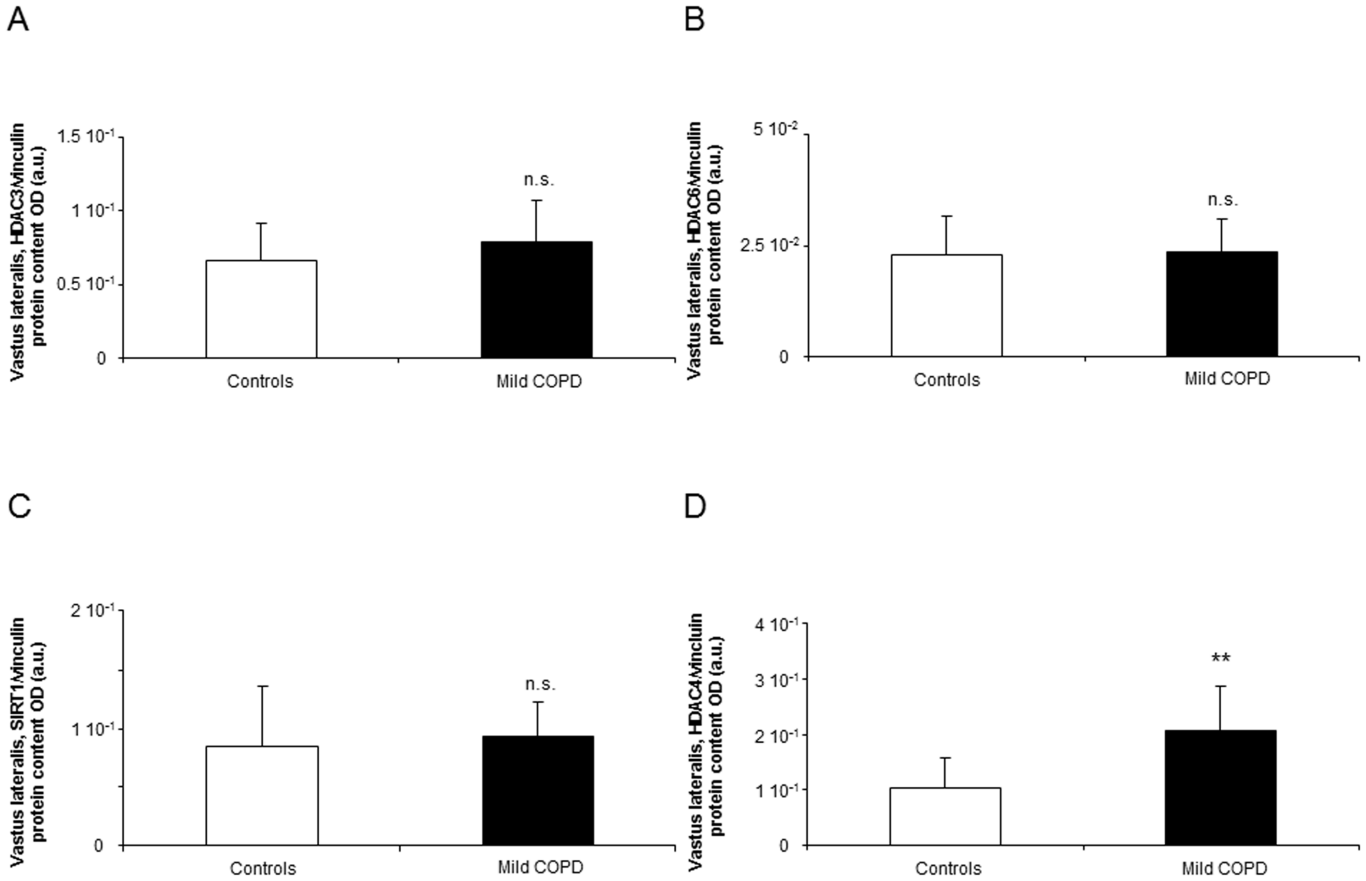
Protein levels of HDACs shown to play a role in muscle dysfunction in muscles of both patients and controls. Mean values and standard deviation of protein HDAC3/vinculin protein loading control (A) HDAC6/vinculin protein loading control as measured in optical densities (OD) using arbitrary units (a.u.) (B), and SIRT1/vinculin protein loading control (C) levels in the vastus lateralis did not differ (n.s., non-significant) between the two study groups, while HDAC4/vinculin protein loading control (D) protein levels were significantly greater (**: p<0.01) in the patients than in the controls.

Total protein acetylation levels in muscles did not differ between patients and control subjects (Figure 5A). Expression levels of the HTA p300 did not differ between patients and healthy controls (Figure 5B). Muscle protein levels of HDAC3 and 6 and SIRT1 did not differ between patients and controls (Figures 6A–6C), while levels of HDAC4 were significantly increased in the vastus lateralis of the patients compared to healthy subjects (Figure 6D).

#### Myogenic transcription factors

**Figure 7:**
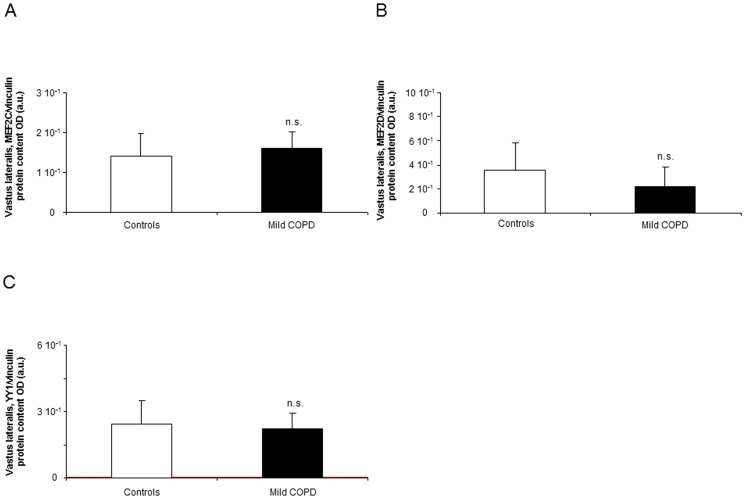
Protein levels of transcription factors shown to be involved in myogenesis and muscle repair in muscles of both patients and controls. Mean values and standard deviation of total MEF2C/vinculin protein loading control as measured in optical densities (OD) using arbitrary units (a.u.) (A), MEF2D/vinculin protein loading control (B), and YY1/vinculin protein loading control (C) levels in the vastus lateralis did not differ (n.s., non-significant) between patients and control subjects.

Compared to controls, protein levels of MEF2C, MEF2D and YY1 did not differ between patients and healthy controls in the limb muscles (Figures 7A–7C, respectively). Significant inverse correlations were observed between protein levels of MEF2C and miR-1 (r = −0.734, p = 0.024) and miR-486 (r = −0.694, p = 0.038) mRNA expression levels.

#### Expression of SUMO

**Figure 8:**
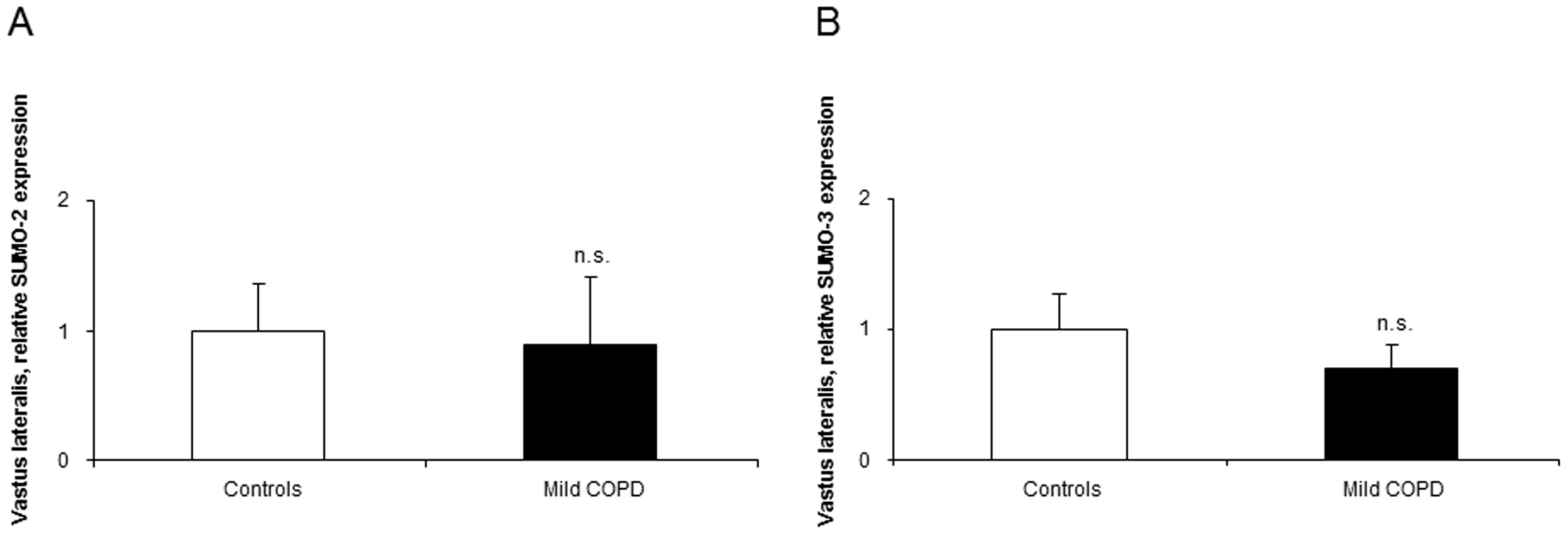
mRNA expression of SUMO-2 and SUMO-3 in the vastus lateralis of mild COPD patients and controls. Mean values and standard deviation (relative expression) of SUMO-2 (A) and SUMO-3 (B) in the vastus lateralis did not differ (n.s., non-significant) between patients and control subjects.

The mRNA expression levels of SUMO2 and SUMO3 did not differ between patients and controls in the vastus lateralis (Figures 8A and 8B, respectively).

### Muscle structure

#### Fiber type composition

In the limb muscles, no significant differences were observed in either fiber type proportions or sizes between patients and controls (Table 4 and Figure S2 in File S1).

**Table 4:** Fiber type composition of all the study subjects.

	Controls N = 13	Mild COPD N = 13
**Muscle fiber type composition**		
**Type I fibers, percentages**	41 (6)	39 (7)
**Type II fibers, percentages**	59 (6)	61 (7)
**Type I fibers, CSA (µm^2^)**	2736 (867)	2543 (604)
**Type II fibers, CSA (µm^2^)**	3010 (939)	2758 (578)

Values are expressed as mean (standard deviation).

*Abbreviations*: CSA, cross-sectional area, µm, micrometer.

## Discussion

As far as we are concerned this is the first study in which a variety of epigenetic events has been explored in the lower limb muscles of patients with mild COPD. Despite that differences in the epigenetic profile of the vastus lateralis in the patients were not strikingly different from that encountered in the healthy subjects, a few differences and significant correlations between the analyzed markers were indeed observed. On these grounds, the study hypothesis was confirmed to a great extent.

The main findings in the current study are that compared to healthy controls, patients with COPD and preserved body composition exhibited clinical and functional signs of mild airway obstruction and emphysema (decreased carbon monoxide transfer). Moreover, the patients also exhibited a moderately reduced exercise capacity as measured by maximal exercise test on a cycloergometer and six-minute walking test together with a mild reduction in quadriceps muscle force as determined by voluntary maneuvers. Additionally, signs of chronic illness were also detected in the blood of the patients as shown by increased levels of fibrinogen and globular sedimentation velocity parameters compared to levels in the healthy controls. Importantly, in the vastus lateralis, a significant rise in the expression levels of miR-1 was observed in the patients compared to those in the healthy controls. Furthermore, significant positive correlations were found between FEV_1_ and quadriceps force and levels of miR-1 among the mild COPD patients. Finally, protein levels of HDAC4 were also increased in the lower limb muscle of the patients compared to those in the control subjects. Other epigenetic parameters or muscle type composition did not significantly differ between patients and healthy controls.

Interestingly, the mild COPD patients already exhibited a significant reduction in their exercise capacity as well as in muscle force generation of the quadriceps. These findings are in line with previous reports in which moderate muscle weakness was also shown to occur in COPD patients at early stages of their disease [2]–[4], [50]. Moreover, in one of the studies [50], muscle dysfunction did not seem to correlate with physical inactivity in patients with very early COPD (GOLD stage I). Additionally, blood parameters that reveal the presence of a chronic disease were also significantly increased in the patients as similarly reported in a previous investigation [51]. Taken together, these are relevant clinical findings that warrant attention as identification of these patients in clinical settings are a target for early therapeutic interventions that may delay the course of the disease, while concomitantly improving the patients' quality of life and prognosis. These are also important reasons that warrant the need to focus on the study of COPD patients at early stages of their disease.

Despite the relevance of DNA methylation in cell fate and development, no significant differences were observed in this epigenetic parameter between patients and healthy controls. It is likely that the balance between DNA methylation and demethylation plays a relevant role in muscle development during myogenesis but not in differentiated skeletal muscle cells. In view of these findings it would be possible to conclude that DNA methylation does not seem to contribute to muscle dysfunction in COPD patients, at least in mild COPD.

In the current investigation, a significant rise in the expression levels of miR-1 was observed in the vastus lateralis of the patients compared to control subjects. In keeping with, a recent study conducted by our group (unpublished observations) showed that miR-1 expression was also upregulated in the lower limb muscles of patients with moderate and severe COPD and preserved body composition. Nevertheless, miR-1 expression together with other muscle-specific microRNAs was downregulated in the vastus lateralis of patients with severe COPD and pronounced muscle wasting as well as in the diaphragm muscle of patients with moderate-to-severe COPD (unpublished observations). Independently of the classic transcription factors, muscle development and repair after injury are also regulated by muscle-specific microRNAs that target different signaling pathways [25], [26], [52]. As such, miR-1 promotes myotube formation and participates in the innervation process of the myofibers. Moreover, miR-1 may also exert its actions on the muscle fibers through several mechanisms such as the insulin-like growth factor (IGF)-1 signal transduction cascade [53] or through induction of HDAC4 expression, which may further promote muscle cell proliferation and differentiation [18], [20], [53]. Indeed, in the present study, HDAC4 protein levels were also shown to be increased in the vastus lateralis of the patients compared to healthy controls. In keeping with, the lower limb muscles of severe COPD patients with relatively preserved body composition also exhibited a rise in HDAC4 expression [20]. Futhermore, in a previous investigation of our group (unpublished observations), HADC4 levels were also shown to be increased in the diaphragm of patients with moderate-to-severe COPD and normal body composition, but not in the vastus lateralis of patients with advanced COPD. Taken together, these findings suggest that HDAC4 expression is likely to be upregulated in the muscles of patients with relatively preserved body composition and muscle mass, regardless of the airway obstruction. They may also indicate that HDAC4 probably plays a major role in muscle repair and mass maintenance in patients with a well-preserved muscle compartment. Whether miR-1 and HDAC4 may drive the maintenance of muscle mass in respiratory and limb muscles of patients with COPD remain to be elucidated though it could be an interesting question for future research.

Importantly, significant positive correlations were found between miR-1 expression levels in the lower limb muscle of the patients and the degree of the airway obstruction as measured by FEV_1_ and the force generated by the quadriceps muscle. Again these findings reinforce the concept that miR-1 is likely to be involved in the maintenance of muscle mass and function in COPD at early stages of the disease. In addition, in the muscles of the patients, significant correlations were also found between miR-1 levels and the expression of miR-486 and miR-133, which are highly involved in muscle proliferation and differentiation. These observations may also indicate that in skeletal muscles, microRNAs possibly operate in a network fashion in order to ensure a continuous muscle repair process after injury, at least in patients with well-preserved body composition.

Muscle mass loss may be the result of hyperacetylation of proteins through several mechanisms that render proteins more prone to catabolism by ubiquitin-ligase activity of several HTAs, and by dissociation of proteins from cellular chaperones [28]–[31], [54]. Among several HTAs, the nuclear cofactor p300 has been shown to regulate muscle differentiation and wasting in several experimental in vivo and in vitro models [28]–[31], [54], [55]. Moreover, in several experimental models of muscle wasting [28], [29], [31], [54], levels of HDAC3, HDAC6, and SIRT-1 were also shown to be decreased in muscles. In the current investigation, no differences were detected in p300, HDAC3, HDAC6 or SIRT-1 expression levels between patients and healthy controls. It is likely that hyperacetylation drives muscle wasting in more advanced COPD, especially when additional factors such as deconditioning and nutritional abnormalities take place in the patients, and negatively influence muscle phenotype. Taken together, these results imply the existence of a rather complex epigenetic regulation of muscle mass maintenance in patients with COPD than a simple up or downregulation of genes and pathways involved in myogenesis.

Premature senescence of primary myogenic cells and other cell models [32], [33] was shown to be related to the accumulation of SUMO ligases [32]. Moreover, premature satellite cell senescence through SUMO ligases also underlies the pathophysiology of muscular dystrophies [33]. In the current study, expression levels of SUMO-2 and -3 did not differ between patients and healthy controls. It is possible to conclude from these findings that premature senescence does not seem to be a relevant mechanism of muscle dysfunction in patients with COPD.

### Study limitations

A first limitation in the current investigation has to do with the fairly small number of subjects studied. However, on the basis of the relatively “invasive” nature of this investigation, with patients with very mild or mild COPD patients and healthy controls undergoing a muscle biopsy from the vastus lateralis, we felt discouraged to recruit more patients and controls for the purpose of this investigation. Moreover, as abovementioned in the corresponding Methods section, the sample size of both patient and control populations was calculated on the basis of formerly published studies by our group and other investigators, where similar physiological and biological approaches were used in both mild COPD patients and healthy control subjects [3]–[7], [10], [45]–[47], [56]. Therefore, it is reasoned herein that the relatively small number of patients and controls included was sufficient to demonstrate the current study hypothesis.

A second limitation in the study is related to its relatively descriptive nature. However, on the basis of currently available literature, it is not possible to answer the questions that the study addresses. Therefore, the present investigation represents a first attempt to assess the pattern of epigenetic events that take place in the lower limb muscles of patients with mild COPD. Most of the investigations conducted so far have focused on the study of patients with advanced COPD, in whom most of the molecular events underlying COPD muscle dysfunction have been identified. Nonetheless, elucidation of the events that may take place in muscles of patients with very mild COPD is of relevance as therapeutic measures could be applied at earlier stages of the disease.

### Conclusions

Moderate skeletal muscle dysfunction is a relevant feature in patients with mild COPD and preserved body composition. Several epigenetic events are differentially expressed in the limb muscles of these patients, probably as an attempt to counterbalance the underlying mechanisms that alter muscle function and mass. The study of patients at early stages of their disease is of interest as they are a target for timely therapeutic interventions that may slow down the course of the disease and prevent the deleterious effects of major comorbidities.

## Supporting Information

File S1 **Detailed methodologies.** **Figure S1 in File S1. Representative immunoblot of vinculin protein content as the loading control in the vastus lateralis of both patients and healthy controls. Figure S2 in File S1. Representative immunohistochemical preparations corresponding to the staining of type II fibers in the vastus lateralis of a healthy control subject and a patient with mild COPD.**
(DOCX)
