# Supplementary material for: Do Epigenetic Events Take Place in the Vastus Lateralis of Patients with Mild Chronic Obstructive Pulmonary Disease?
Source: PLoS One. 2014 Jul 11;9(7):e102296. doi: 10.1371/journal.pone.0102296 (PMC4094498; doi:10.1371/journal.pone.0102296)
Supplement: File S1 — Detailed methodologies. Figure S1 in File S1. Representative immunoblot of vinculin protein content as the loading control in the vastus lateralis of both patients and healthy controls. Figure S2 in File S1. Representative immunohistochemical preparations corresponding to the staining of type II fibers in the vastus lateralis of a healthy control subject and a patient with mild COPD. (DOCX) [file pone.0102296.s001.docx]

**Supporting information**

**do EPIGENETIC events take place in the VASTUS LATERALIS of patients with mild Chronic Obstructive Pulmonary Disease?**

**Ester Puig-Vilanova, Pilar Ausin, Juana Martinez-Llorens, Joaquim Gea****, and Esther Barreiro**

**METHODS**

**Study subjects**

Thirteen patients with stable COPD and 13 age-matched sedentary controls were recruited. Specimens from the vastus lateralis were obtained from all subjects on an out-patient basis. COPD patients were recruited from the COPD [1–4] Clinic at Hospital del Mar (Barcelona) and the control subjects were recruited from the general population (patients’ relatives or friends) at Hospital del Mar. Smoking history was similar between patients and healthy controls. All patients were on bronchodilators. They were clinically stable at the time of the study, without episodes of exacerbation or oral steroid treatment in the previous four months. None of them presented significant comorbidities. All groups of individuals were Caucasian. Moreover, in the present investigation, the sedentary control subjects were also involved in another study aimed at investigating the epigenetic profile in limb muscles of patients with severe COPD and muscle wasting (unpublished observations, submitted).

Exclusion criteria for COPD patients and control subjects included other chronic respiratory (asthma) or cardiovascular disorders, acute exacerbations in the last 3 months, limiting osteoarticular condition, chronic metabolic diseases including diabetes, suspected para-neoplastic or myopathic syndromes, and/or treatment with drugs known to alter muscle structure and/or function including systemic corticosteroids. COPD patients and healthy controls were qualified as sedentary after being specifically inquired about whether they were conducting any regular outdoor physical activity, going regularly to the gymnasium, or participating in any specific training program.

The current investigation was designed in accordance with both the ethical standards on human experimentation in our institutions and the World Medical Association guidelines (Helsinki Declaration of 2008) for research on human beings. Approval was obtained from the institutional Ethics Committees on Human Investigation (*Hospital del Mar,* Barcelona). Informed written consent was obtained from all individuals.

**Anthropometrical and Functional Assessment**

Anthropometrical evaluation included BMI and determination of the FFMI by bioelectrical impedance [5]. Nutritional parameters were also evaluated through conventional blood tests.

Lung function was evaluated through determination of spirometric values, static lung volumes, diffusion capacity, and blood gases using standard procedures and reference values by Roca *et al* [6–8].

Quadriceps muscle strength was evaluated in both patients and controls by isometric maximum voluntary contraction (QMVC) of the dominant lower limb as formerly described [9,10]. Patients were seated with both trunk and thigh fixed on a rigid support of an exercise platform (Domyos HGH 050, Decathlon, Lille, France). The highest value from three brief reproducible maneuvers (<5% variability among them) was accepted as the MVC.

**Muscle biopsies and blood samples**

*Vastus lateralis biopsies*. Muscle samples were obtained from the quadriceps muscle (vastus lateralis) of both groups of patients and control subjects using the open muscle biopsy technique, as described previously [5,11]. Samples were 60-80 mg size in average.

Muscle sample specimens were always cleaned out of any blood contamination with saline. They were immediately frozen in liquid nitrogen and stored in the –80ºC freezer (under permanent alarm control) for further analysis or immersed in an alcohol-formol bath for 2h to be thereafter embedded in paraffin. Frozen tissues were used for immunoblotting techniques, while paraffin-embedded tissues were used for the assessment of myosin heavy chain isoforms (immunohistochemical analysis). All subjects were prevented from doing any potentially exhausting physical exercise 10 to 14 days before coming to the hospital to undergo the surgical procedures.

Blood samples were drawn at 8:00 am after an overnight fasting period in both patients and healthy controls.

**Molecular biology analyses**

*DNA isolation***.** Total DNA was isolated from vastus lateralis muscle of all study subjects using QIAmp DNA Mini Kit (QiAgen, Redwood City, CA, USA) [12], following the manufacturer’s protocol of DNA purification from tissues, and without the use of RNase A. Total DNA obtained from muscles was quantified using a spectrophotometer (NanoDrop, Thermo Scientific, Wilmington, DE, USA).

*Quantification of Methylated DNA using enzyme-linked immunosorbent assay (ELISA)-based immunoassay.* Global 5-methylcytosine (5-mC) in DNA was quantified in the vastus lateralis of both patients and healthy controls using MethylFlash Methylated DNA Quantification Colorimetric Kit (Epigentek, Farmingdale, NY, USA) following the precise manufacturer’s instructions and previous studies [13]. Briefly, 80 microliters of Binding Solution were poured into each plate well. Subsequently, 100 nanograms of each DNA sample and positive and negative controls were loaded in duplicates onto the assigned plate wells and covered with sealing tape. The plate was then incubated at 37º for 90 minutes, washed and incubated again with 50 microliters of a specific capture antibody for 1 hour at room temperature. After several washes, 50 microliters of a specific detection antibody was added and the plate was incubated at room temperature for 30 minutes. The plate was then washed several times, and 50 microliters of enhanced solution was poured into each plate well, which was incubated again at room temperature for 30 minutes. Finally, after several washes, 100 microliters of developing solution was added, and the process was stopped using a specific stop solution. Absorbances were read at 450 nm in a microplate spectrophotometer (Microplate reader M680, Bio-Rad, Hercules, CA, USA) using a reference filter of 655 nm. A standard curve was generated and sample concentrations were calculated based on the manufacturer’s instructions. The minimum detectable concentration of methylated DNA in the samples was set to be 0.2 ng of methylated DNA. Data are expressed as the percentage of total methylated DNA to total DNA in the muscle samples.

*RNA isolation***.** Total RNA was first isolated from snap-frozen skeletal muscles using Trizol reagent following the manufacturer’s protocol (Life technologies, Carlsbad, CA, USA). Total RNA concentrations were determined photometrically using the NanoDrop 1000 (Thermo Scientific, Waltham, MA, USA).

*MicroRNA and mRNA reverse transcription (RT)***.** MicroRNA RT was performed using TaqMan microRNA assays (Life Technologies) following the manufacturer’s instructions. First-strand cDNA was generated from mRNA using oligo(dT)_12-18_ primers and the Super-Script III reverse transcriptase following the manufacturer’s instructions (Life technologies).

*Real time-PCR amplification (qRT-PCR)*. TaqMan based qPCR reactions were performed using the ABI PRISM 7900HT Sequence Detector System (Applied BioSystems, Foster City, CA, USA) together with a commercially available predesigned microRNA assay, primers, and probes as shown in Tables 1 and 2. Taqman microRNA assay for small nuclear RNA U6 (snU6) was used to normalize the miRNAs amplifications, whereas the housekeeping gene glyceraldehyde-3-phosphate dehydrogenase (GAPDH) served as the endogenous control for mRNA gene expression. MicroRNA and mRNA data were collected and subsequently analyzed using the SDS Relative Quantification Software version 2.1 (Applied BioSystems), in which the comparative C_T_ method (2^-ΔΔCT^) for relative quantification was employed [14]. Results in the figures are expressed as the expression of fold change relative to mean value of the control group, which was equal to 1.

*Immunoblotting of 1D electrophoresis*. Protein levels of the different molecular markers analyzed in the study were explored by means of immunoblotting procedures as previously described [5,11,15–17]. Briefly, frozen muscle samples from the vastus lateralis muscles of both patients and control subjects were homogenized in a buffer containing HEPES 50 mM, NaCl 150 mM, NaF 100 mM, Na pyrophosphate 10 mM, EDTA 5 mM, Triton-X 0.5%, leupeptin 2 μg/ml, PMSF 100 μg/ml, aprotinin 2 μg/ml and pepstatin A 10 μg/ml. The entire procedures were always conducted at 4ºC. Protein levels in crude homogenates were spectrophotometrically determined with the Bradford method using triplicates in each case and bovine serum albumin (BSA) as the standard (Bio-Rad protein reagent, Bio-Rad Inc., Hercules, CA, USA). The final protein concentration in each sample was calculated from at least two Bradford measurements that were almost identical. Equal amounts of total protein (ranging from 20 to 100 micrograms, depending on the antigen and antibody) from crude muscle homogenates were always loaded onto the gels, as well as identical sample volumes/lanes. For the purpose of comparisons among the different groups of COPD patients and controls, muscle sample specimens were always run together and kept in the same order.

Two fresh 10-well mini-gels were always simultaneously loaded for each of the antigens and run together in the same mini-cell box. Experiments were confirmed at least twice for all the antigens analyzed in the investigation. Fresh gels were specifically loaded for each of the antigens in most of cases. However, in a very few cases, antigens were identified from stripped membranes.

# Proteins were then separated by electrophoresis, transferred to polyvinylidene difluoride (PVDF) membranes, blocked with bovine serum albumin and incubated overnight with selective primary antibodies. Protein levels of HDACs, HATs, and myogenic transcription factors were identified using specific primary antibodies: HDAC3 (anti-HDAC3 antibody, Santa Cruz Biotechnology, Santa Cruz, CA, USA), HDAC6 (anti-HDAC6 antibody, Epigentek, Farmingdale, NY, USA), HDAC4 (anti-HDAC4 antibody, Santa Cruz), NAD-dependent protein deacetylase sirtuin-1 (SIRT1) (anti-SIRT1 antibody, ProteinTech Group Inc., Chicago, IL, USA), myocyte enhancer factor (MEF)2C (anti-MEF2C antibody, Santa Cruz), MEF2D (anti-MEF2D antibody, Santa Cruz), Yin Yang (YY)1 (anti-YY1 antibody, Santa Cruz), and vinculin (anti-vinculin antibody, Santa Cruz). Antigens from all samples were detected with horseradish peroxidase (HRP)-conjugated secondary antibodies and a chemiluminescence kit. For each of the antigens, samples from the different groups were always detected in the same picture under identical exposure times.

PVDF membranes were scanned with the Molecular Imager Chemidoc XRS System (Bio–Rad Laboratories, Hercules, CA, USA) using the software Quantity One version 4.6.5 (Bio–Rad Laboratories). Optical densities of specific proteins were quantified using the software Image Lab version 2.0.1 (Bio-Rad Laboratories). In order to validate equal protein loading among various lanes, SDS-PAGE gels were stained with Coomassie Blue, and the cytoskeletal protein vinculin (117 kDa, Figure S1) was used as the protein loading controls in all the immunoblots. Final optical densities obtained in each specific group of subjects corresponded to the mean values of the different samples (lanes) of each of the antigens studied, which were normalized to the optical densities of the loading control (vinculin) for each antigen and experimental group.

Standard stripping methodologies were employed when detection of the antigens required the loading of a relatively greater amount of total muscle protein. Very briefly, membranes were stripped of primary and secondary antibodies through one 30-minute washes with a stripping solution (25 mM glycine, pH 2.0 and 1% SDS) followed by two consecutive 10-minute washes containing phosphate buffered saline with tween (PBST) at room temperature. Membranes were blocked with bovine serum albumin and reincubated with primary and secondary antibodies following the procedures described above.

*Muscle fiber counts and morphometry.* On 3-micrometer muscle paraffin-embedded sections from vastus lateralis muscles of all study groups, MyHC-I and –II isoforms were identified using anti-MyHC-I (clone MHC, Biogenesis Inc., Poole, England, UK) and anti-MyHC-II antibodies (clone MY-32, Sigma, Saint Louis, MO), respectively, as published elsewhere [5,11,17]. The cross-sectional area, mean least diameter, and proportions of type I and type II fibers were assessed using a light microscope (Olympus, Series BX50F3, Olympus Optical Co., Hamburg, Germany) coupled with an image-digitizing camera (Pixera Studio, version 1.0.4, Pixera Corporation, Los Gatos, CA, USA) and a morphometry program (NIH Image, version 1.60, Scion Corporation, Frederick, MD, USA). At least 100 fibers were measured and counted in each muscle specimen from both study groups.

**Statistical Analysis**

Data are expressed as mean (standard deviation). Comparisons of physiological, clinical, molecular and structural variables between the two study groups were analyzed using the Student’s *T- test*. Correlations between clinical, physiological and biological variables were explored using the Pearson’s correlation coefficient among both groups of patients. A level of significance of *P*≤ 0.05 was established.

The sample size chosen was based on previous studies [5,11,15–24], where very similar approaches were employed and on assumptions of 80% power to detect an improvement of more than 20% in measured outcomes at a level of significance of *P*≤ 0.05.

Reference List

1. Miravitlles M, Soler-Cataluna JJ, Calle M, Molina J, Almagro P, et al. (2012) Spanish COPD Guidelines (GesEPOC): pharmacological treatment of stable COPD. Spanish Society of Pulmonology and Thoracic Surgery. Arch Bronconeumol 48: 247-257. S0300-2896(12)00115-9 [pii];10.1016/j.arbres.2012.04.001 [doi].

2. Miravitlles M, Calle M, Soler-Cataluna JJ (2012) Clinical phenotypes of COPD: identification, definition and implications for guidelines. Arch Bronconeumol 48: 86-98. S0300-2896(11)00342-5 [pii];10.1016/j.arbres.2011.10.007 [doi].

3. Miravitlles M, Soler-Cataluna JJ, Calle M, Molina J, Almagro P, et al. (2014) Spanish Guideline for COPD (GesEPOC). Update 2014. Arch Bronconeumol 50 Suppl 1: 1-16. S0300-2896(14)70070-5 [pii];10.1016/S0300-2896(14)70070-5 [doi].

4. Rieger-Reyes C, Garcia-Tirado FJ, Rubio-Galan FJ, Marin-Trigo JM (2013) Classification of Chronic Obstructive Pulmonary Disease Severity According to the New Global Initiative for Chronic Obstructive Lung Disease 2011 Guidelines: COPD Assessment Test Versus Modified Medical Research Council Scale. Arch Bronconeumol. S0300-2896(13)00306-2 [pii];10.1016/j.arbres.2013.09.014 [doi].

5. Fermoselle C, Rabinovich R, Ausin P, Puig-Vilanova E, Coronell C, et al. (2012) Does oxidative stress modulate limb muscle atrophy in severe COPD patients? Eur Respir J 40: 851-862. 09031936.00137211 [pii];10.1183/09031936.00137211 [doi].

6. Roca J, Sanchis J, Agusti-Vidal A, Segarra F, Navajas D, et al. (1986) Spirometric reference values from a Mediterranean population. Bull Eur Physiopathol Respir 22: 217-224.

7. Roca J, Rodriguez-Roisin R, Cobo E, Burgos F, Perez J, et al. (1990) Single-breath carbon monoxide diffusing capacity prediction equations from a Mediterranean population. Am Rev Respir Dis 141: 1026-1032.

8. Roca J, Burgos F, Barbera JA, Sunyer J, Rodriguez-Roisin R, et al. (1998) Prediction equations for plethysmographic lung volumes. Respir Med 92: 454-460.

9. Swallow EB, Reyes D, Hopkinson NS, Man WD, Porcher R, et al. (2007) Quadriceps strength predicts mortality in patients with moderate to severe chronic obstructive pulmonary disease. Thorax 62: 115-120.

10. van den Borst B, Slot IG, Hellwig VA, Vosse BA, Kelders MC, et al. (2013) Loss of quadriceps muscle oxidative phenotype and decreased endurance in patients with mild-to-moderate COPD. J Appl Physiol (1985) 114: 1319-1328. japplphysiol.00508.2012 [pii];10.1152/japplphysiol.00508.2012 [doi].

11. Barreiro E, Peinado VI, Galdiz JB, Ferrer E, Marin-Corral J, et al. (2010) Cigarette smoke-induced oxidative stress: A role in chronic obstructive pulmonary disease skeletal muscle dysfunction. Am J Respir Crit Care Med 182: 477-488.

12. Andreu AL, Martinez R, Marti R, Garcia-Arumi E (2009) Quantification of mitochondrial DNA copy number: pre-analytical factors. Mitochondrion 9: 242-246. S1567-7249(09)00039-7 [pii];10.1016/j.mito.2009.02.006 [doi].

13. Hadoux J, Favier J, Scoazec JY, Leboulleux S, Al GA, et al. (2014) SDHB mutations are associated with response to temozolomide in patients with metastatic pheochromocytoma or paraganglioma. Int J Cancer . 10.1002/ijc.28913 [doi].

14. Livak KJ, Schmittgen TD (2001) Analysis of relative gene expression data using real-time quantitative PCR and the 2(-Delta Delta C(T)) Method. Methods 25: 402-408. 10.1006/meth.2001.1262 [doi];S1046-2023(01)91262-9 [pii].

15. Barreiro E, de la Puente B, Minguella J, Corominas JM, Serrano S, et al. (2005) Oxidative stress and respiratory muscle dysfunction in severe chronic obstructive pulmonary disease. Am J Respir Crit Care Med 171: 1116-1124. 200407-887OC [pii];10.1164/rccm.200407-887OC [doi].

16. Barreiro E, Rabinovich R, Marin-Corral J, Barbera JA, Gea J, et al. (2009) Chronic endurance exercise induces quadriceps nitrosative stress in patients with severe COPD. Thorax 64: 13-19.

17. Marin-Corral J, Minguella J, Ramirez-Sarmiento AL, Hussain SN, Gea J, et al. (2009) Oxidised proteins and superoxide anion production in the diaphragm of severe COPD patients. Eur Respir J 33: 1309-1319.

18. Barreiro E, Schols AM, Polkey MI, Galdiz JB, Gosker HR, et al. (2008) Cytokine profile in quadriceps muscles of patients with severe COPD. Thorax 63: 100-107.

19. Barreiro E, Ferrer D, Sanchez F, Minguella J, Marin-Corral J, et al. (2011) Inflammatory cells and apoptosis in respiratory and limb muscles of patients with COPD. J Appl Physiol 111: 808-817. japplphysiol.01017.2010 [pii];10.1152/japplphysiol.01017.2010 [doi].

20. Donaldson A, Natanek SA, Lewis A, Man WD, Hopkinson NS, et al. (2013) Increased skeletal muscle-specific microRNA in the blood of patients with COPD. Thorax 68: 1140-1149. thoraxjnl-2012-203129 [pii];10.1136/thoraxjnl-2012-203129 [doi].

21. Koechlin C, Couillard A, Simar D, Cristol JP, Bellet H, et al. (2004) Does oxidative stress alter quadriceps endurance in chronic obstructive pulmonary disease? Am J Respir Crit Care Med 169: 1022-1027.

22. Lewis A, Riddoch-Contreras J, Natanek SA, Donaldson A, Man WD, et al. (2012) Downregulation of the serum response factor/miR-1 axis in the quadriceps of patients with COPD. Thorax 67: 26-34. thoraxjnl-2011-200309 [pii];10.1136/thoraxjnl-2011-200309 [doi].

23. Natanek SA, Riddoch-Contreras J, Marsh GS, Hopkinson NS, Man WD, et al. (2011) Yin Yang 1 expression and localisation in quadriceps muscle in COPD. Arch Bronconeumol 47: 296-302. S0300-2896(11)00090-1 [pii];10.1016/j.arbres.2011.02.015 [doi].

24. Ortega F, Toral J, Cejudo P, Villagomez R, Sanchez H, et al. (2002) Comparison of effects of strength and endurance training in patients with chronic obstructive pulmonary disease. Am J Respir Crit Care Med 166: 669-674. 10.1164/rccm.2107081 [doi].

**
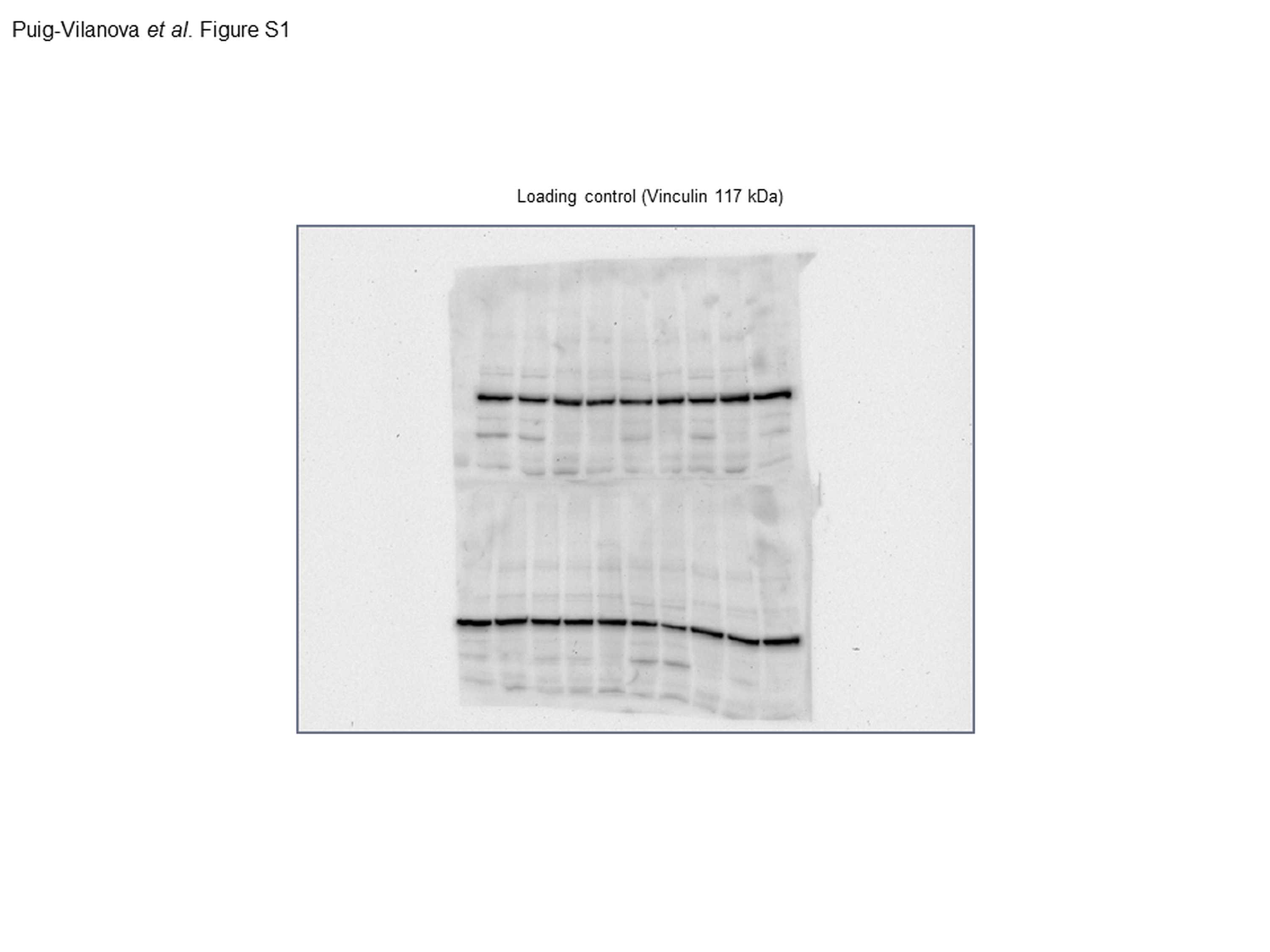

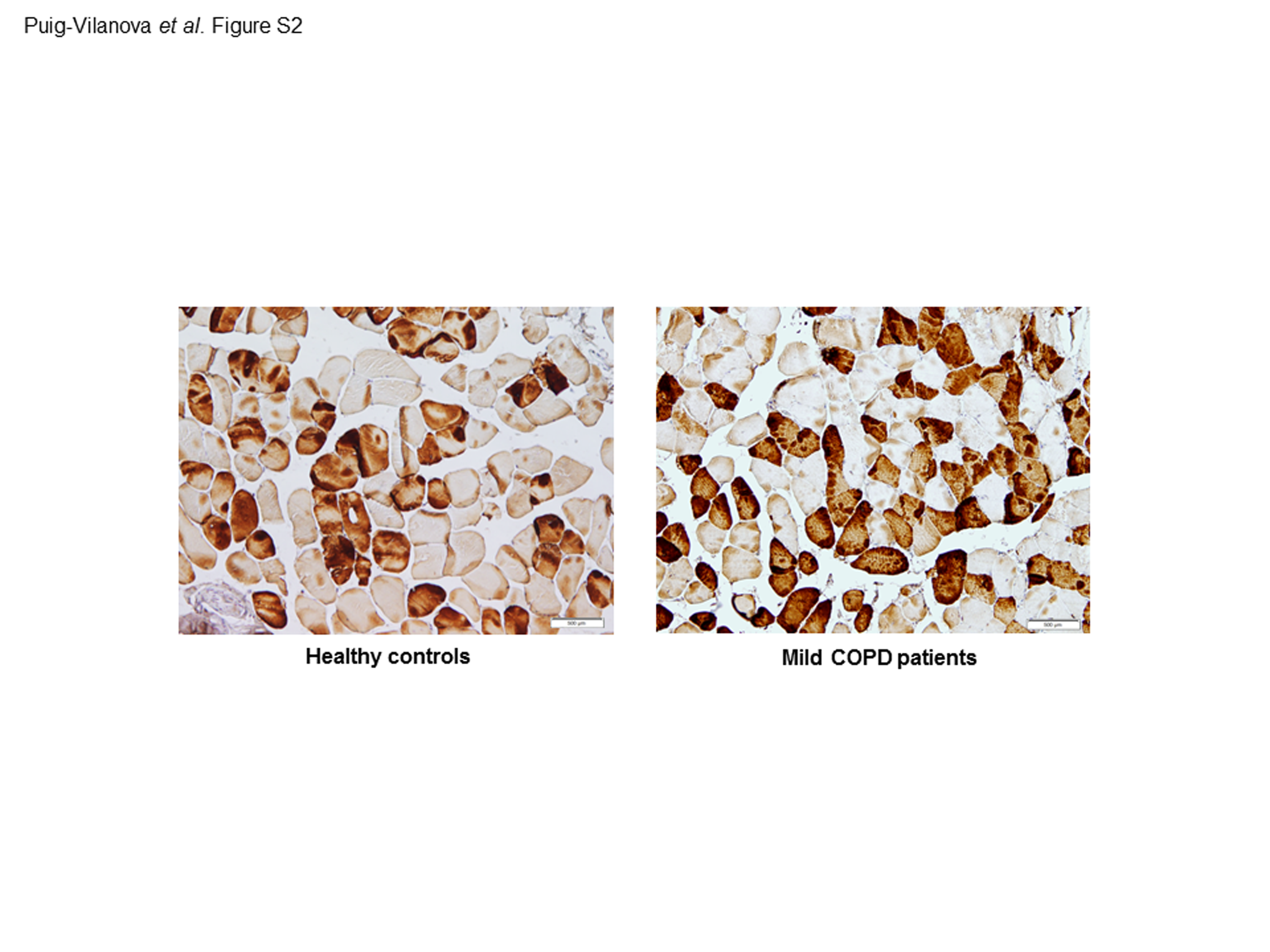
**
